# Development and Clinical Applications of Therapeutic Cancer Vaccines with Individualized and Shared Neoantigens

**DOI:** 10.3390/vaccines12070717

**Published:** 2024-06-27

**Authors:** Qing Hao, Yuhang Long, Yi Yang, Yiqi Deng, Zhenyu Ding, Li Yang, Yang Shu, Heng Xu

**Affiliations:** 1 State Key Laboratory of Biotherapy and Cancer Center, Department of Biotherapy, West China Hospital, Sichuan University, Chengdu 610041, China; haoqing@stu.scu.edu.cn (Q.H.); long_yuhang@stu.scu.edu.cn (Y.L.); yangyi666@stu.scu.edu.cn (Y.Y.); dengyq@scu.edu.cn (Y.D.); dingzhenyu@scu.edu.cn (Z.D.); yl_tracy@scu.edu.cn (L.Y.); 2 Colorectal Cancer Center, Department of General Surgery, West China Hospital, Sichuan University, Chengdu 610041, China; 3 Gastric Cancer Center, Department of General Surgery, West China Hospital, Sichuan University, Chengdu 610041, China; 4 Institute of General Surgery, West China Hospital, Sichuan University, Chengdu 610041, China; 5 Research Center of Clinical Laboratory Medicine, Department of Laboratory Medicine, West China Hospital, Sichuan University, Chengdu 610041, China

**Keywords:** neoantigen, therapeutic cancer vaccines, cancer immunotherapy

## Abstract

Neoantigens, presented as peptides on the surfaces of cancer cells, have recently been proposed as optimal targets for immunotherapy in clinical practice. The promising outcomes of neoantigen-based cancer vaccines have inspired enthusiasm for their broader clinical applications. However, the individualized tumor-specific antigens (TSA) entail considerable costs and time due to the variable immunogenicity and response rates of these neoantigens-based vaccines, influenced by factors such as neoantigen response, vaccine types, and combination therapy. Given the crucial role of neoantigen efficacy, a number of bioinformatics algorithms and pipelines have been developed to improve the accuracy rate of prediction through considering a series of factors involving in HLA-peptide-TCR complex formation, including peptide presentation, HLA-peptide affinity, and TCR recognition. On the other hand, shared neoantigens, originating from driver mutations at hot mutation spots (e.g., KRAS^G12D^), offer a promising and ideal target for the development of therapeutic cancer vaccines. A series of clinical practices have established the efficacy of these vaccines in patients with distinct HLA haplotypes. Moreover, increasing evidence demonstrated that a combination of tumor associated antigens (TAAs) and neoantigens can also improve the prognosis, thus expand the repertoire of shared neoantigens for cancer vaccines. In this review, we provide an overview of the complex process involved in identifying personalized neoantigens, their clinical applications, advances in vaccine technology, and explore the therapeutic potential of shared neoantigen strategies.

## 1. Introduction

Cancer is largely attributed to the accumulation of somatic genomic alterations, which not only promotes malignant cell outgrowth, proliferation, and survival but also enables the immune system to identify and attack cancer cells by distinguishing them as ‘foreign’ [1]. Different types of genomic alterations were identified in tumor cells, including single nucleotide variants (SNV), copy number alterations, and structural variants, thus disrupting normal cellular functions and leading to the malignant transformation of cells. Crucially, these alterations have the potential to generate novel epitopes through protein translation, proteasome degradation and Major Histocompatibility Complex (MHC) class I presentation. These new antigenic epitopes, recognized as ‘non-self’ by the host, are referred to as tumor-specific antigens (TSAs) or neoantigens. Their exclusive presence in malignant tissues triggers a series of tumor-specific immune responses [2], thereby endowing neoantigens as valuable targets for cancer treatment.

Recently, extensive clinical studies have underscored the antitumor activity of neoantigen-based vaccines in patients across various cancers, including the severe cancer types (e.g., pancreatic cancer) [3,4,5,6,7,8,9,10]. Although in phase I/II trials with small sample size, cancer vaccines have exhibited great success to reduce the tumor volume and prolong the overall survival of cancer patients, thus were considered as the next immunotherapy frontier [11]. However, this personalized therapeutic approach has substantial limitations, including the high cost and time-consuming synthesis processes, a lack of consensus on the best practices for neoantigen discovery, an unclear optimal vaccine delivery scheme, and challenges in transferring between individuals. To achieve robust clinical efficacy, further development and investigation are necessary. Notably, in addressing the major rate-limiting step in the manufacture of individualized vaccines, the development of shared vaccines targeting recurrent drive mutation-derived neoantigens presents a viable solution for enhancing clinical efficacy in an efficient and economical manner [12]. 

Herein, we provide an overview of the complex procedures involved in identifying personalized neoantigens, outline the clinical development landscape of therapeutic neoantigen-based cancer vaccines, and discuss the clinical benefits of combining neoantigen-based vaccines with other therapies. Furthermore, we explore the therapeutic promise of research into shared neoantigen strategies and their clinical applications.

## 2. Prediction and Selection of Neoantigens

### 2.1. Source of Neoantigen

**Figure 1:**
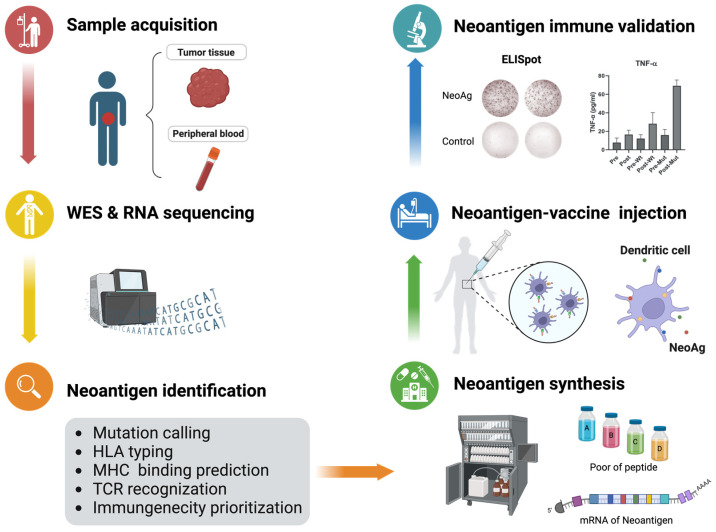
The full design of neoantigen vaccine. Patient tumor samples and peripheral blood mononuclear cells are isolated for DNA and RNA sequencing, followed by the initiation of the neoantigen prediction process. In this workflow, tumor-specific mutations are first identified, along with patient HLA typing. The binding of mutated peptides to HLA alleles and their potential to elicit T cell responses are predicted and prioritized for neoantigen screening. Using the chosen vaccine platform (such as mRNA, peptides, or dendritic cells), personalized neoantigen vaccines are produced on demand under Good Manufacturing Practice (GMP) conditions. After immunization, the immunogenicity of the selected neoantigens is validated using immunological assays with the patient’s peripheral blood T cells. WES: Whole exome sequencing.

High-throughput sequencing technology and bioinformatics analysis have now enabled the in silico prediction of potential neoantigens, revolutionizing our approach to cancer research (Figure 1). In conventional practices, tumor tissues and paired normal samples (e.g., peripheral blood) are collected and analyzed by whole exome sequencing (WES) to identify protein-altering somatic mutations, comprising single-nucleotide variants (SNVs), short insertions and deletions/insertion of nucleotides (indels). In addition, transcriptomic sequencing-based analysis not only provides insights into the expression levels of mutated genes and helps to further mutation verification, but also detects fusion genes and tumor specific splicing. Somatic mutations occurring in the coding regions could serve as the mechanism for the ‘foreignness’ of cancer cells and alter protein sequences in various ways. Given their abundance in many cancers, SNVs are the earliest targets used to identify neoantigens and remain a primary focus within this field. Although only a single amino acid substitution, SNV-derived neoantigens are capable of eliciting the antitumor immune responses [3,4,5,13]. In contrast, Indels can result in the in-frame or frameshift translations of the open reading frame, producing polypeptide sequences with little or no homology to the wild-type counterpart, thereby enhancing immunogenicity. The ‘dissimilarity’ of antigens to their wild-type counterpart has been utilized as a predictive indicator to evaluate recognition by host immunity [14,15]. Furthermore, fusion genes, which link two unrelated genes by intra- or inter-chromosomal rearrangements, are another potential resource of highly immunogenic neoantigens [16]. Although a series of recurrent fusion genes have been identified in multiple cancer types, particularly common in leukemia [11,17,18], the vast majority of fusion genes appear to be individual occurrences, since gene fusions are considered relatively rare events [19,20].

**Figure 2:**
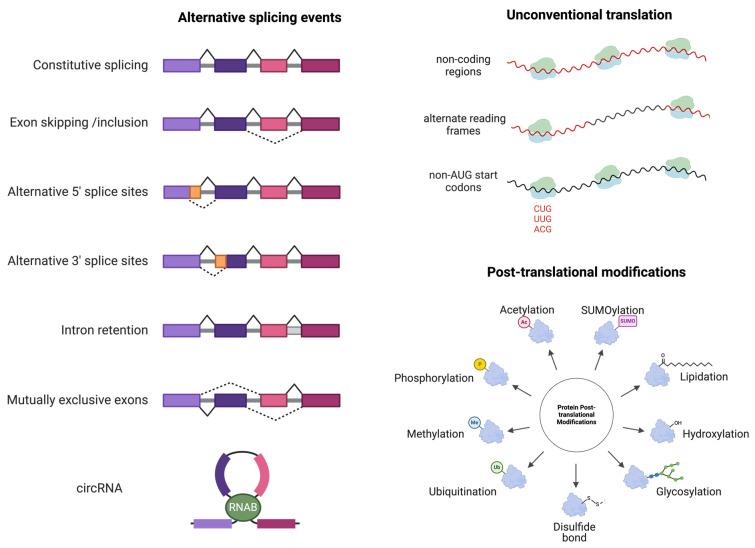
Cryptic peptide derived from non-canonical transcription and translation. Non-canonical transcription is an alternative splicing event. Non-canonical translation often includes: translation of ‘non-translated’ regions, translation of non-canonical reading frames, and translation of non-AUG start codons. Changes in post-translational modification patterns may also lead to the production of cancer-specific neoantigens.

Confronted with the problem of low mutation burden in many cancer types [21], investigators are looking beyond the coding region to cryptic peptides derived from non-canonical transcription and translation [22,23] (Figure 2). These cryptic peptides, emerging from ‘non-translated’ region, non-canonical reading frames, and initiation at non-AUG start codons, introduce novel protein sequences with minimal overlap with their regular counterparts [22]. Recent studies have demonstrated that cryptic antigens constitute a significant portion of the cancer-associated immunopeptidome [24,25]. Specifically, alternative splicing events, including back-splicing circularization that generates circular RNAs, have been identified as sources of cryptic neoantigens, expanding the landscape of tumor-specific antigens [23,26,27,28]. Alternative splicing allows a single gene to produce multiple protein isoforms [29], and is more prevalent in the various types of cancers compared to that in the normal tissues [26]. Such an expansion offers promising new opportunities for neoantigen-based therapy [30]. Furthermore, altered patterns of post-translational modifications, influenced by cancer-specific pathways, might affect the immunological foreignness which potentially leads to the creation of cancer-specific neoantigens [31,32]. However, the cryptic neoantigens and their full potential in immunotherapy remain to be fully elucidated, suggesting a clear direction for future research in this promising area.

Recently, pioneering studies have shown that intra-tumor bacteria are an intrinsic part of the tumor microenvironment (TME) across various human cancer types, residing intracellularly within the cytoplasm of both tumor cells and immune cells [33]. This discovery underscores the complex interplay between microbial presence and cancer biology. Furthermore, T cell response elicited by bacteria could cross-react with tumor antigens, suggested that homologous epitopes shared by both the bacteria and tumors can contribute to anti-tumor immunity [34,35]. Such findings suggest that exposing microbial epitopes through the elimination of intra-tumor bacteria could provide alternative tumor epitopes for cancer immunotherapy. Building on this concept, a pivotal study has shed light on this approach by delivering antibiotics encapsulated in liposomes to target intra-tumor bacteria, eliciting an anti-tumor CD8^+^ T cell response activated by microbial-derived neoantigens [36]. Although these cross-reactive T cell responses have been initially uncovered in mouse models, they present an innovative strategy for harnessing the immune system against cancer, thereby expanding the repertoire of neoantigens available for cancer immunotherapy.

### 2.2. Prediction of Neoantigen

The workflows for neoantigen discovery have been extensively reviewed by several research groups [37,38,39,40,41]. Therefore, herein we briefly overview the key processes of neoantigen prediction and summarize recent advancements in this field. 

Several teams have released their open-source neoantigen prediction pipelines, including MuPeXI [42], INTEGRATE-NEO [43], NeoPredPipe [44], pTuneos [45], ASNEO [46], NeoFuse [47], pVACtools [48], QBRC [49], Seq2Neo [50], nextNEOpi [51], ImmuneMirror [52]. Most of these tools are one-stop solution for neoantigen identification, starting with raw sequences data from DNA and/or RNA. The latest software, spanning from 2020 to the present, are detailed in Table 1. SNVs, indels, and gene fusions are included as the main sources in the majority of tools, such as nextNEOpi, pVACtools, Seq2Neo, etc. Specifically, the QBRC neoantigen calling pipeline introduces a novel indicator to evaluating neoantigen clonal balance, named the Cauchy–Schwarz index of Neoantigens (CSiN). This immunogenicity index is also included in the nextNEOpi tool.

However, in most cases, these prediction pipelines employ only one or two tools during the key analysis processes of neoantigen prediction, involving mutation calling, HLA typing, and MHC-peptide binding, which could lead to high rates of false positives. In contrast, nextNEOpi and pVACtools stand out by applying multiple tools for these tasks. Employing multi-algorithm consensus approaches likely yields more accurate results, as distinct algorithms typically utilize different detection strategies.

## 3. Presentation by MHC Molecular

The pivotal step for in silico neoantigen prediction relies on the production and presentation of peptides by MHC-I and MHC-II alleles. A number of prediction tools have been developed, including netMHCpan [53], MixMHCpred [54,55], MHCflurry [56], and HLAthena [57], which based on different functions [58,59]. Notably, netMHCpan and MixMHCpred are capable of predicting MHC class I and II-restricted peptide. In recent years, one of the major advances in MHC-peptide binding prediction has been the training of networks on eluted ligands data detected by immunoprecipitation and followed liquid chromatography-tandem mass spectrometry (LC-MS/MS), which directly provides endogenously processed and presented peptides from cells [53,56,57]. In addition, MHCflurry, EDGE [60] and MARIA [61] consider protease cleavage and flanking sequences as presentation features. Besides these established tools, we summarize the novel methods developed in the past two years for MHC-I and MHC-II binding prediction (Table 2).

Computational algorithms for MHC-peptide binding prediction have two categories: allele-specific and pan-specific [68]. In allele-specific methods, a separate model is trained for each MHC allele, which inevitably leads to underperformance for rare MHC alleles with insufficient ligand data. This issue can be addressed by pan-specific MHC-peptide binding predictor or tools like netMHCpan use the homology MHC sequence to infer potential binding properties. Pan-specific methods integrate information about MHC alleles and peptides into a single model, enabling simultaneously learning the binding properties of all MHC alleles. Tools such as DeepMHCI and DeepMHCII utilize this approach.

Furthermore, the stability of MHC-peptide complexes (pMHC) serves as another critical indicator for immunogenicity prediction in cancer vaccines. For instance, a high-throughput stability screening method utilize a standard real-time (RT)-PCR instrument to determine temperature denaturation curves and assess stability [69], while a neural network predictor (i.e., NetMHCstabpan) was also developed [70]. Both studies recommend integrating the stability information with MHC-peptide binding to improve the selection of neoantigens in cancer vaccines.

## 4. Recognition by TCR

The recognition and interaction of TCR-pMHC are the crucial determinants of immunogenicity. The TCR-pMHC prediction models can be broadly categorized into three groups: (a), Similarity-Based TCR Profiling Model: These models, such as TCRdist [71], GIANA [72], and GLIPH2 [73], utilize a similarity-weighted Hamming distance to cluster a set of related TCRs and visualize their specific binding motif; (b), Restricted peptide TCR recognition model: Models represented by netTCR [74], netTCR-2.0 [75], TCRex [76] predict TCR sequence recognition for specific peptides, such as viral and few cancer epitopes; (c) Broad-scope TCR-pMHC interaction prediction model: Models like SETE [77], ERGO-II [78], and pMTnet [79], are designed to predict interactions for given peptides and TCRs, crucial for neoantigen prediction pipelines. For example, pMTnet utilize peptide, MHC alleles and CDR3 sequences of TCRβ as inputs to predict the binding rank of each TCR-pMHC, while ERGO-II integrates multiple levels of information, including epitopes, MHC alleles, T cell type, CDR3α and CDR3β amino acid, and corresponding V and J genes.

Up to now, TCR recognition has made substantial strides. The development of novel tools that utilize various machine learning algorithms to enhance the prediction of TCR-pMHC interactions is detailed in Table 3. Among these algorithms, particularly noteworthy is TEIM [80], which use convolutional neural networks (CNN) to learn local interaction between CDR3βs and peptides, and Panpep [81], which adopts neural Turing machines (NTM) to improve TCR binding specificity prediction for any peptide, especially neoantigens or exogenous peptides. Unfortunately, current pMHC-TCR interaction predictors are still at a preliminary level, primarily due to the limited validated pMHC-TCR interaction data for adequate training, and a poorly understanding of the underlying binding mechanisms. This underscores the importance of continued research and data collection to refine these predictive models.

## 5. Neoantigen and Immunotherapies

T cell responses against neoantigens have emerged as the core effectors of cancer therapeutic strategies. Therapeutic cancer vaccines not only amplify pre-existing endogenous T cell responses and but also induce de novo ones. Beyond neoantigen-based cancer vaccines, neoantigen-specific T cells also drive the efficacy of immune checkpoint blockade (ICB) [99,100] and adoptive T cell therapies [101]. Additionally, the success of ICB is closely correlated with the tumor mutation burden (TMB) across a variety of cancers [100,102,103]. However, it is the tumor neoantigen burden (TNB)—a measure directly employed in neoantigen evaluation—that may serve as a superior biomarker for immunotherapy outcomes [104,105,106]. TNB provides a more direct assessment of immunogenic potential, thereby potentially improving the predictive accuracy of therapeutic responses.

### 5.1. Neoantigen-Based Cancer Vaccines

The earliest cancer vaccines were developed to prevent liver and cervical cancers, which are caused by hepatitis B virus (HBV) [107] and human papillomavirus (HPV), respectively. Nowadays, the focus of cancer vaccines has shifted to a therapeutic strategy against personalized neoantigens. These therapeutic cancer vaccines have entered clinical trials, including dendritic cell (DC)-based [7,108], mRNA-based [4,10,109], and peptide-based vaccines [3,5,6,8,13] (Table 4). In general, these vaccines are designed to combine approximately 20 neoantigens across multiple complementary categories, such as class I-restricted and II-restricted, clone and subclone. This strategic design is aimed at mitigating the risk of off-target effects and addressing immune escape, thereby enhancing efficacy and safety. Additionally, several ongoing clinical trials are summarized in the Supplementary Table S1, highlighting the continued efforts to refine and expand the use of neoantigen-based vaccines. For some “cold” cancer types (e.g., glioblastoma) with limited somatic mutation rate, combination of neoantigen and tumor associated antigens (TAAs) were also used to increase the potential targets.

#### 5.1.1. mRNA-Based Vaccines

mRNA vaccines emerge as a promising alternative to traditional vaccine methods, showing encouraging outcomes in infectious diseases, like COVID-19 [114,115] and various cancer types [4,10,109]. They boast numerous benefits, including high potency, rapid development, and low-cost production [116], due to the high yields of in vitro transcription (IVT) reactions and advanced industrial setups [117]. Given that naked mRNA is rapidly degraded by extracellular RNases and is unable to penetrate cell membranes, effective delivery systems are essential for the successful applications of these vaccines [118]. A common approach is to encapsulate the mRNA within lipid nanoparticles (LNPs), that are tiny spheres designed to protect the mRNA molecules and their delivery into cells and tissues [119,120]. 

In the realm of cancer vaccines, neoantigen-based mRNA vaccines offer several additional advantages: (a) a single mRNA can incorporate multiple distinct neoantigens, thereby increasing the vaccine’s breadth and potency; (b) mRNAs can encode full-length or long-kmer neoantigen, containing multiple neoepitopes without MHC-restriction. Sahin et al. demonstrated its feasibility in a phase I clinical trial of personalized neoantigen vaccines. In this study, 13 patients with stage III and IV melanoma received a therapeutic mRNA vaccine targeting up to 10 neoantigens. These 10 neoantigens were engineered into two synthetic RNA molecules, each encoding five linker-connected 27mer neoantigens. Neoantigen-specific CD4+ T cell and CD8+ T cell responses are observed in all patients, which led to a sustained progression-free survival [4]. 

A recent mRNA neoantigen vaccine developed by BioNTech has demonstrated inspiring clinical benefit [10]. Personalized mRNA vaccines were delivered intravenously to patients with pancreatic ductal adenocarcinoma (PDAC). Each mRNA strand encodes up to 10 MHC-I and 10 MHC-II-restricted neoantigens, and encapsulated in LNPs. In the vaccine-responder patients, a prolonged recurrence-free survival (RFS) was observed compared to non-responder patients, suggesting mRNA-based neoantigen vaccine could induce potent antitumor effects.

#### 5.1.2. Peptide-Based Vaccines

In recent years, peptide-based therapeutic cancer vaccines targeting neoantigens have achieved significant clinical benefits [3,5,6,8,13]. Due to peptide vaccines’ relative ease of manufacturing, low toxicity, and high chemical stability during storage [121], peptide-based vaccines are the preferred choice for most trials [122]. However, a major limitation of this approach is its inherently low immunogenicity, which hampers broader clinical application [123]. To address this issue, researchers have developed several strategies to boost the immunological response. In studies conducted over the last few decades, the size of the vaccinated peptides has proved critical for stimulating protective antitumor immunity. The optimal design involves using synthetic 15–30 mer long neoantigens peptides rather than 8–11 mer short peptides that represent the core epitopes of CD8+ T cells. Short peptides can be direct presentation by all nucleated cells, leading to suboptimal T cells activation. In contrast, long peptides require processing by professional antigen-presenting cells (APCs) residing in draining lymph nodes (dLNs), thereby ensuring the sustained expansion of effector CD8+ T cells [124]. Moreover, long peptides engage both CD4+ and CD8+ T cell responses, prolonging the duration of antigen presentation [125]. Furthermore, the coadministration of appropriate adjuvants via the subcutaneous route, including Cytokines such as GM-CSF (granulocyte–macrophage colony-stimulating factor), toll-like receptor (TLR) agonist like poly-ICLC (lysine and carboxymethylcellulose) and CpG, could induce powerful antitumor vaccine responses [121]. 

Ott et al. launched peptide-based neoantigens vaccines in six high-risk melanoma patients [3]. As a pioneering work in personalized therapeutic neoantigen clinical trial, they designed their peptide vaccine: synthesized long peptides with poly-ICLC to target up to 20 neoantigens per individual. Four of these patients remained recurrence-free 25 months post-vaccination, while two relapse achieved complete tumor regression following subsequent anti-PD-1 therapy. In long-term follow-ups to four years, these antitumor T cell responses induced by neoantigens demonstrated strong efficacy, suggesting neoantigen-specific T cells converted into a memory phenotype and provide long-lasting protection [126]. The same peptide-based vaccine scheme also induces neoantigen-specific T cell responses in glioblastoma patients [5]. In another glioblastoma trial, Hilf reposted their personalized vaccination phase I results [13]. Their vaccines that had poly-ICLC and GM-CSF adjuvants displayed favorable safety and strong immunogenicity, extending the median progression-free survival to 14.2 months, and overall survival to 29.0 months.

The immunogenicity of peptide vaccines can be further enhanced through developing novel deliver systems, especially nanoparticle platform [127]. Nanoparticles can co-deliver peptides and adjuvants, enhancing their delivery to dLNs, and thus promoting DC internalization, which is necessary for long peptide-based vaccines [128,129]. For example, a high-density lipoprotein-mimicking nanodisc was manufactured to co-deliver neoantigen peptides and CpG, and it elicited up to 47-fold greater frequencies of neoantigen-specific T cells than free CpG plus neoantigen peptides [130]. The co-delivery scheme can be implemented by structure-based programming of Lymph-node-targeted amphiphile (Amph) vaccines [131]. The components of Amph vaccines (neoantigens and adjuvants) are modified with diacyl lipids, exhibiting both hydrophilic and lipophilic characteristics and thus can bind with albumin in the plasma. These albumins act as carrier molecules that guide the vaccine to the lymph nodes, enhancing lymph node accumulation and efficient delivery into APCs. 

These Amph vaccines have been implemented pancreatic and colorectal cancer, where neoantigens derived with KRAS G12D and G12R [132]. The phase 1 study shows that among 25 patients, 84% exhibited KRAS-specific T cell responses, and 24% observed tumor biomarker clearance.

#### 5.1.3. DC-Based Vaccines

As the most potent APCs, DC can be generated, loaded, and administered to stimulate robust antitumor responses in vivo, thus becoming optimal cell population for vaccination purposes [133]. As mentioned above, both mRNA and peptide-based vaccines elicit neoantigen-specific T cells that rely on the uptake of neoantigens by endogenous DCs, a process that is relatively inefficient. DC-based vaccines, however, can efficiently accomplish this step ex vivo through pulsing or transfection, thereby maximizing their efficacy [134]. Autologous DCs are initially isolated from patients, subsequently loaded with neoantigens and allowed to mature under optimal conditions. Once matured, these DCs, now equipped with substantial immune-stimulating capabilities, are reintroduced into the patient.

The personalized neoantigen DC vaccines have been proved safe and effective in humans as early as 2015. Carreno and his colleagues first reported the clinical results of a small phase I trial in advanced melanoma patients [108]. They used DC pulsing with synthetic neoantigens peptides and observed the increased neoantigen-specific T cells in breadth and diversity. In 2021, a single-arm cohort study initiated by West China Hospital applied the personalized neoantigen DC-based vaccines in patients with advanced recurrent lung cancer [7]. They vaccinated subcutaneously 12 heavily treated patients with metastatic lung cancer using synthetic neoantigens peptides pulsing DC. All patients tolerated and responded well to this therapy, with an overall response rate (ORR) of 25%, a disease control rate (DCR) of 75%, a median progression-free survival (mPFS) of 5.5 months, and a median overall survival (mOS) of 7.9 months. These findings demonstrated the efficacy of neoantigen DC-based vaccines in cancer patients for the first time.

Recently, Liau reposted a phase III clinical trial of the autologous tumor lysate-loaded DC neoantigen vaccines (DCVax-L) in glioblastoma patients [135]. The study enrolled 331 patients with newly diagnosed glioblastoma (nGBM) and recurrent glioblastoma (rGBM), with 232 randomized to the DCVax-L group and 99 to the placebo group. Survival rates after 60 months from randomized therapy was 13.0% in the DCVax-L group vs. 5.7% in control, confirming that DCVax-L treatment greatly enhances long-term survival for nGBM patients. For patients with rGBM, the median OS from relapse was 13.2 months in the DCVax-L group compared to 7.8 months in the control cohort. This is the first trial proven to extend the survival of patients with rGBM through neoantigen therapy.

### 5.2. TCR-T

Adoptive T cell transfer therapy has been significantly reshaped during the development of neoantigens, evolving towards the generation of engineering T cells equipped with neoantigen-specific T cell receptors (TCRs). This innovative approach, known as TCR-T therapy, bears similarities to CAR-T therapy in that it involves modifying a patient’s autologous T lymphocytes ex vivo. Known TCR sequences that target specific neoantigens are introduced before reinfusing the modified cells back into the patient for cancer treatment. Several clinical trials implementing TCR-T therapy have yielded encouraging results in treating melanomas, synovial cell sarcoma, ovarian, and pancreatic cancers [136,137,138,139,140]. This approach has a number of advantages: including high antigen sensitivity and near-physiological signaling, which enhance tumor cell detection and killing while also improving T cell persistence [141].

Initially, Robbins reported a series of clinical trial using TCR-T therapy that targeted the HLA-A*02:01-restricted NY-ESO-1 antigen, a well-known type of TAA (also classified as a cancer/testis antigen) [137,138,139]. NY-ESO-1 is expressed in 10% to 50% of metastatic melanomas, breast, prostate, thyroid, and ovarian cancers, as well as 80% of synovial cell sarcomas, with no expression in normal adult tissues except the testis [138]. In these trials, objective clinical responses were observed in 11 of 18 patients with synovial cell sarcoma, 11 of 20 patients with melanoma, 16 of 20 patients with multiple myeloma. The success of these trials confirmed the safety and effectiveness of TCR-T therapy and inspired further development of TCR-T targeting personalized neoantigens.

For TCR-T targeting neoantigens, the process involves the following steps: neoantigens are first identified by the prediction workflow, followed by the synthesis of peptides to stimulate CD8+ T cells to obtain neoantigen-specific T cells; these cells are then sequenced to identify their TCRs, enabling the generation of neoantigen-specific TCR-engineered T cells. The feasibility of this preparation protocol was demonstrated in a pilot study within just 2 weeks [136]. One notable application involved an HLA-C*08:02-restricted TCR-T targeting the mutant KRAS p.G12D in a patient with metastatic pancreatic cancer, where regression of visceral metastases was observed, with the response enduring beyond 6 months [140].

### 5.3. Combination Therapies

Combining neoantigen vaccines with other immunotherapies—including ICB, adoptive T cell transfers, surgical excision, radiotherapy or chemotherapy—could enhance therapeutic efficacy for cancer treatment, particularly in treating advanced or aggressive cancers. A recent trial involving a neoantigen vaccine for pancreatic cancer demonstrated significant clinical benefits, which supports this perspective. This trial implemented a multifaceted treatment approach that included surgical excision, ICB, neoantigen vaccines, and four-drug chemotherapy regimen [10]. Furthermore, neoantigen vaccines have been effectively used in some clinical trials to delay cancer recurrence post-surgery, yielding impressive results in esophageal cancer, as indicated in trial NCT05023928.

The synergy between vaccines and ICB treatments can be explained by their complementary mechanisms. Cancer therapeutic vaccines have the potential to overcome ICB resistance and boost the effectiveness of ICB in combination immunotherapy [142,143]. Conversely, ICB has been shown to improve the immunogenicity of neoantigen vaccines and increase the tumor vaccine response rate, as demonstrated in colorectal cancer [144]. Furthermore, an inherent challenge with therapeutic neoantigen vaccines is that the antitumor T cells they elicit may become dysfunctional and exhausted when facing a high tumor burden. Induction therapy with ICB might help mitigate this issue, improving the overall effectiveness and durability of the immune response [40].

## 6. Shared Vaccine

Although personalized neoantigen vaccines have demonstrated clinical benefits in various cancers, the time and economic costs of their implementation are unavoidable challenges. To address these limitations, many research groups are simultaneously exploring ‘off-the-shelf’ immunotherapy options. These alternatives, such as shared neoantigen or tumor-associated antigen (TAA) vaccines, offer a more immediate and cost-effective solution. For instance, in some studies, researchers inject TAA vaccines into patients until release of their neoantigen vaccine, which typically takes at least 3 months [4,13]. This approach could provide on-going immune protection against cancer during the waiting period.

### 6.1. A Special Class of Neoantigen: Shared Neoantigen

Neoantigens derived from driver mutations in oncogenes or tumor suppressors are attractive targets for immunotherapy. Unlike most ‘passengers’ somatic mutations that do not contribute to oncogenesis, vaccines based on shared neoantigens offer three significant advantages.

Firstly, most driver mutations occur in the early stage of tumor development and are considered trunk mutations. Given the high degree of heterogeneity in tumors, trunk mutations exist in the majority of tumor cells, making them more suitable targets for vaccine targets. Secondly, while a substantial portion of patients initially respond to immunotherapy, many subsequently develop mechanisms of immune evasion. These mechanisms include deficits in the antigen presentation machinery, loss of neoantigens, or exploitation of alternate immune checkpoint pathways [145]. However, the loss of driver mutations can impair oncogenic pathways, suggesting that selecting neoantigen from driver mutations may reduce the likelihood of immune escape to some extent. Thirdly, some driver mutations recur with high frequencies across different types of cancers, offering the potential for shared targets among patients. This pan-cancer applicability enhances the utility of neoantigen-based vaccines, potentially simplifying the development of broad-spectrum oncological treatments. Further details on the specific neoantigens discussed are provided in Table 5.

#### 6.1.1. KRAS

KRAS driver mutations, mostly occurred on 12th or 13th amino acid, which are commonly found in pancreatic ductal adenocarcinoma (PDAC) and colorectal cancer (CRC), present as attractive targets for immunotherapy. These mutations are essential for tumor survival and exhibit constitutive activation throughout the progression of the disease [146,147]. Recently, a variety of immunotherapies targeting KRAS mutations have emerged.

Adoptive HLA C*08:02-restricted KRAS p.G12D-specific T cells transfer therapy has demonstrated the objective regression in patients with metastatic PDAC and CRC [140,148]. Six months after the engineered T cells transfer, the response was still ongoing, and made up more than 2% of all circulating peripheral-blood T cell [140]. However, TCR-T scheme is restricted to MHC alleles, and is not conducive to the further application between patients. A clinical trial has been conducted using long peptide vaccines targeting KRAS p.G12D/R mutations in 20 PDAC and 5 CRC patients, where 84% exhibited specific T cell responses to KRAS p.G12D/R and observed tumor biomarker responses [132]. The vaccine effectively co-delivered both neoantigens and adjuvants to the APCs. After internalization, neoantigens are presented by the patient’s MHC molecules, featuring simplified manufacturing and off-the-shelf availability.

#### 6.1.2. TP53

TP53 is one of the most frequently mutated genes in human cancer. The loss of p53 protein can lead to the occurrence of cancer, and the development of drugs and therapies targeting TP53 mutations has always been a focal point [149]. Recently, Rosenberg and his team have recognized an immunogenic neoantigen (HMTEVVRHC) originating from TP53 p.R175H mutation within the HLA-A*02:01 context in a patient with metastatic colorectal cancer. This discovery led to the identification of specific T cells and TCR sequences that target this neoantigen [150], laying the foundation for subsequent adoptive cell transfer therapy that expresses the same MHC allele and mutation.

Building on this foundational work, they conducted TCR-T therapy on a patient with chemotherapy-resistant breast cancer. By transducing her own peripheral blood T cells with an allogeneic HLA-A*02:01-restricted TCR specific for TP53 p.R175H [151], they observed objective tumor regression that lasted for six months. Simultaneously, they also treated 12 patients with chemotherapy-resistant epithelial cancer, employing adoptive transfer of ex vivo-expanded autologous tumor-infiltrating lymphocytes (TIL) without any genetic engineering. However, this trial achieved limited clinical responses, with only 2 out of 12 patients showing partial responses. Furthermore, targeting the same mutation can also be engineered into a bispecific antibody, H2-scDb, to stimulate T cell killing of TP53-mutant tumor cells [152]. These outcomes highlight the potentially greater efficacy of engineered T cells targeting mutant TP53, compared to the unmodified TIL approach. 

#### 6.1.3. IDH1

The IDH gene, responsible for encoding isocitrate dehydrogenase, can undergo mutations on 132th amino acid that disrupt cellular metabolism and potentially induce aberrant DNA methylation [153]. Such mutations have been identified across a spectrum of tumor types and are an early and decisive event in the development of gliomas. The IDH1 p.R132H mutation recurs in over 70% of diffuse grade II and grade III gliomas. Therefore, Schumacher et al. made neoantigen peptide library targeting this mutation and demonstrated that, the long peptide p123-142 (GWVKPIIIGHHAYGDQYRAT) is immunogenic neoantigen that can elicit class II-restricted CD4+ specific-T cell response in HLA-DRB1*01:01 context [154].

#### 6.1.4. EGFR

The epidermal growth factor receptor (EGFR) is a transmembrane protein that possesses cytoplasmic kinase activity and conveys critical growth factor signals from the extracellular environment to the cell [155]. Considering that over 60% of non-small cell lung carcinomas (NSCLCs) exhibit EGFR expression, it has emerged as a crucial target for cancer therapy [155]. 

Lizee and his colleagues conducted a phase I trial of personalized neoantigen peptide vaccines in 24 stage III/IV NSCLC patients who had previously progressed following multiple conventional therapies, including surgery, radiation, chemotherapy, and tyrosine kinase inhibitors (TKIs) [156]. Notably, all seven responders in the trial harbored EGFR mutations, from which two highly shared immunogenic neoantigens were identified: KITDFGRAK from p.L858R, restricted by HLA-A*11:01, and LTSTVQLIM from T790M, restricted by HLA-C*C15:02. Furthermore, it is estimated that approximately 15% of Asian NSCLC patients with EGFR mutations exhibit both the HLA-A*11:01 and p.L858R mutations, pointing to a significant subset of patients who could benefit from tailored therapeutic strategies [157,158].

#### 6.1.5. PIK3CA

Mutations in PIK3CA (the gene encoding phosphatidylinositol 3-kinase alpha, PI3Kα) are among the most common genetic alterations driving oncogenesis. PIK3CA mutations, including H1047R, H1047L, E545K, and E542K, show high prevalence in cancers such as BRCA, CESC, and COAD, affecting 24%, 20%, and 16% of cases, respectively [159]. Building on this, Chandran established a panel of TCRs that specifically recognize the neoantigen HLA-A*03:01-ALHGGWTTK, derived from the PIK3CA hotspot mutation p.H1047L, highlighting the immunogenicity of the common shared neoantigens in prevalent HLA molecules [160]. Following the panel of TCRs, the team validated their functionality through TCR-T adoptive therapy in a mouse model. Tumor regression exclusively in mice bearing the PIK3CA mutation, whereas those with wild-type PIK3CA did not respond to the treatment. These findings underscore the therapeutic potential of shared neoantigens derived from mutant PIK3CA.

#### 6.1.6. ALK

Anaplastic lymphoma kinase (ALK) rearrangements account for 5–6% of all non-small cell lung cancer (NSCLC) cases and are caused by the fusion of ALK with other partner genes. Although several ALK-targeted tyrosine kinase inhibitors (TKIs) have been approved for patients with ALK-positive (ALK+) NSCLC, achieving complete regression is rare due to the development of resistance to these ALK TKIs. This presents a significant challenge in the long-term management of the disease.

In response to this issue, Professors Roberto and Rafael B. developed a neoantigen vaccine that targets ALK mutations [161]. This innovative approach elicits a strong immune response against ALK, which has shown promising results in preclinical models. Specifically, the vaccine has been successful in eradicating primary tumors in mice and preventing the occurrence of metastatic disease. Additionally, in patients with ALK+ NSCLC, they identified four ALK peptides (AMLDLLHVA, RPRPSQPSSL, IVRCIGVSL, VPRKNITLI) that are presented by HLA-A*02:01 and HLA-B*07:02. The immunogenicity and safety of vaccines targeting these neoantigens were validated in human HLA-transgenic mice, paving the way for the development of a clinical vaccine to treat ALK+ NSCLC.

**Table 5:** Selected immunotherapies targeting shared neoantigens.

Gene	Frequency	Neoantigen Peptides	MHC Restriction	Immunotherapy
KRAS	93% PDAC 50% CRC	G12D-YKLVVVGADGVGKSALTI G12R-YKLVVVGARGVGKSALTI	/	Neoantigen vaccines [132]
		G12D-GADGVGKSA(L)	HLA-C*08:02	TCR-T therapy [140,148]
TP53	20% BRCA	R175H-HMTEVVRHC	HLA-A*02:01	TCR-T therapy [145], bispecific antibody [152]
IDH1	70% GL	R132H-GWVKPIIIGHHAYGDQYRAT	HLA-DRB1*01:01	Neoantigen vaccines [154]
EGFR	60% NSCLC	L858R-KITDFGRAK	HLA-A*11:01	Neoantigen vaccine [156]
		T790M-LTSTVQLIM	HLA-C*C15:02	
PIK3A	24% BRCA 20% CESC 16% COAD	H1047L-ALHGGWTTK	HLA-A*03:01	TCR-T therapy [160]
ALK	5% NSCLC	AMLDLLHVA	HLA-A*02:01	Neoantigen vaccines [161]
		IVRCIGVSL RPRPSQPSSL VPRKNITLI	HLA-B*07:02	

### 6.2. Tumor Associated Antigens

**Figure 3:**
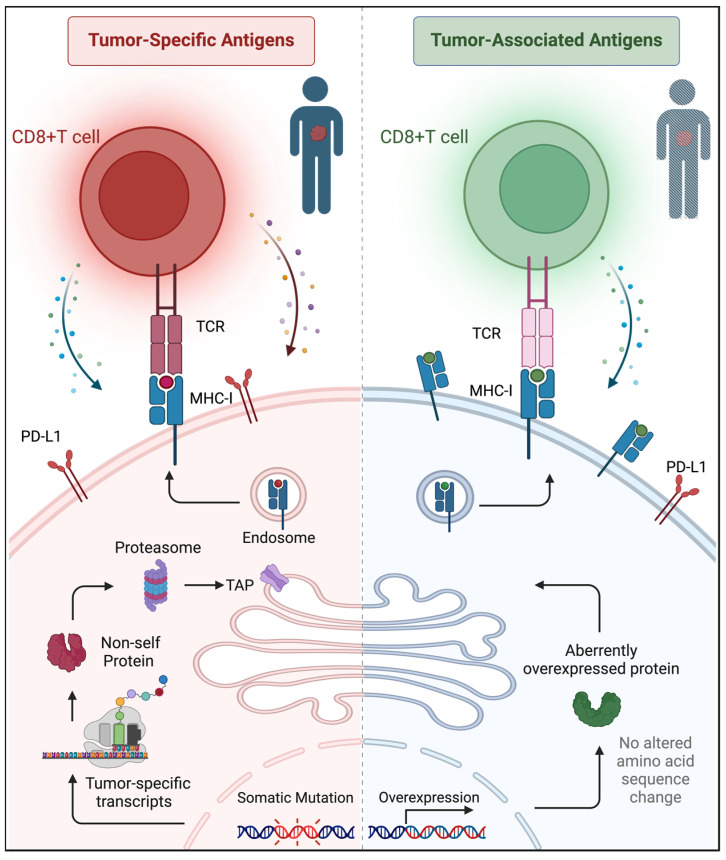
The distinctions between TAA and TSA. TSA arises from altered protein sequences caused by tumor-specific mutations. These alterations can be presented on the tumor cell surface through the endogenous antigen processing pathway, thereby triggering direct cytotoxic responses from the host’s T cells. Conversely, TAA typically originates from genes that are abnormally overexpressed in tumor cells but are either unexpressed or minimally expressed in normal tissues. TAAs are also capable of provoking immune responses, positioning them as promising shared targets for immunotherapy across various cancer types. Furthermore, following the demise of cancer cells, cellular debris is engulfed by neighboring cells, thereby enabling the presentation of TAA and TSA to CD4+ T cells via MHC class II. TAA/TSA-specific T cells are initially activated by antigen-presenting cells (APCs), such as dendritic cells (DCs), which carry these epitopes. This process typically occurs in peripheral lymphoid organs, and the activated T cells subsequently differentiate into cytotoxic T cells, which ultimately infiltrate the tumor and attack the cancer cells.

Therapeutic cancer vaccines have historically targeted tumor associated antigens (TAAs), which, unlike tumor specific antigens, may also be expressed in normal cells/tissues but are aberrantly overexpressed in tumor (Figure 3). TAAs have been identified earlier than tumor specific antigens because they can be detected without high throughput sequencing. Building on this discovery, the initial efforts to develop therapeutic cancer vaccines focused on these aberrantly expressed self-antigens. However, in numerous cancer vaccine trials across various tumor types, most vaccination strategies only induced blood TAA-specific T cell responses; they did not achieve objective clinical benefit [162]. The reasons for this could include: (1) TAA-specific T cells have not proliferated in sufficient numbers to recognize and eliminate tumor cells. (2) The lack of appropriate maturation signals results in T cells that are either unresponsive or produce regulatory T cells with suppressive effects. (3) TAA-specific T cells do not remain within the tumor long enough to effectively kill the malignant cells [163]. Among the few successes, only Provenge^®^ (sipuleucel-T for prostate cancer targeting prostate acid phosphatase) has been approved by the U.S. Food and Drug Administration (FDA). This vaccine has achieved a moderate improvement in treatment outcomes, extending median survival by approximately 4 months [164]. Regrettably, even Sipuleucel-T failed due to its limited efficacy and high cost, reflecting the broader challenges in TAA-based vaccine design including overcoming central or acquired immune tolerance, and achieving a sufficient affinity of T cells towards TAAs. 

As the field of cancer immunotherapy evolves, the development of therapeutic cancer vaccines presents new paradigms toward personalized neoantigen-based vaccine, particularly toward personalized neoantigen-based vaccines. However, the application of TAAs has not been neglected; instead, there is a shift towards utilizing a combination of multiple TAAs. In some trials, TAA vaccines were used to bridge the waiting period during the preparation of neoantigen vaccines [4,13]. Additionally, Adotévi et al. developed a clinical trial using a universal cancer peptide–based vaccine (UCPVax) targeting telomerase reverse transcriptase (TERT), a protein found to be overexpressed in more than 85% of cancers [165]. This vaccine, consisting of two highly selected 15-mer peptides derived from TERT, was administered to 59 patients with refractory advanced NSCLCs, achieving disease control in 21 cases (39%) and a median overall survival of 9.7 months. Moreover, Kjeldsen et al. designed an immunomodulatory vaccine targeting PD-L1 and indoleamine 2,3-dioxygenase (IDO), both critical immunoregulatory molecules in the tumor microenvironment. This vaccine, which includes specific antigens (21-mer peptide DTLLKALLEIASCLEKALQVF from IDO, and 19-mer peptide FMTYWHLLNAFTVTVPKDL from PD-L1), was used in combination with nivolumab to treat patients with metastatic melanoma, achieving an objective response rate of 80% and a complete response rate of 43% [166]. These results highlight the safety and immunogenicity of TAA-based vaccine, suggesting its potential as a significant addition to cancer treatment regimens. However, these trials also reveal limitations. Despite the inclusion of multiple peptides in the vaccine designs, the same peptides were uniformly administered to each patient, likely due to cost considerations. This approach, however, ignored the individualized effects of MHC molecule presentation, which are essential for assessing the immunogenicity of TAA/neoantigens. In light of these limitations, clinical trials utilizing individualized MHC-specific TAA peptides may offer a more significant clinical benefit and demonstrate the potential of personalized immunotherapy to enhance treatment efficacy. 

Moreover, it has been well-established that several cell types (e.g., cancer-associated fibroblasts, and tumor-associated macrophage) in the tumor microenvironment (TME) can facilitate the progression and drug resistance of cancer cells through the secretion of factors or direct interaction [167,168,169], and thus represent a potential therapeutic target for tumor treatment [170,171,172]. With the development and rapid applications of single cell RNA sequencing techniques in investigating the TME, specific cell subtypes that can contribute to tumor progression have been identified, including *SPP1*^+^ macrophage, *FAP*^+^ *GPX3*^+^ cancer associated fibroblast, and *CYP4F3*^+^ monocyte at pan-cancer level [173,174,175]. Consequently, the aberrantly upregulated genes in TME components can also be the potential resource of TAAs. 

## 7. Challenges and Prospects

In recent years, extensive preclinical and clinical research have tested various strategies of neoantigen discovery and vaccine formulations. Immunotherapy targeting neoantigen has achieved impressive effects, but several obstacles must be overcome to elicit potent antitumor response and achieve full clinical benefit. One major challenge is the low response rates of predicted neoantigens in practical clinical applications, where only a few candidate neoantigens were recognized by patient-derived T cells. To address this issue, iterative refinement of software tools using experimental data and optimization of machine learning models for better prediction of MHC-peptide binding affinity and prioritize immunogenicity are recommended. Secondly, the antitumor effects induced by vaccine-specific T cells are often limited due to the suppressive tumor microenvironment. Combining neoantigen vaccines with other immunotherapies could potentially overcome this bottleneck, enhancing therapeutic efficacy. Additionally, the sampling of tumor tissues is difficult to obtained, thus highlighting the importance of sequencing ctDNA from the liquid biopsy for neoantigen prediction and clinical decision-making [176,177,178].

Beyond their application in cancer immunotherapy, neoantigens also play a significant role in explaining susceptibility to autoimmune diseases. For instance, recent studies have shown that carboxyethyl modification of a cysteine residue in integrin αIIb disrupts immune tolerance and generates pathogenic neoantigens. These neoantigens, presented by HLA-DRB1*04, stimulate CD4+ T cell responses and induce the production of autoantibodies, leading to autoimmune diseases such as ankylosing spondylitis (AS) [179].

Finally, the development of ‘off-the-shelf’ strategies provides opportunities for patients with specific cancer type patients harboring recurrent driver mutations. These vaccines offer a cost-effective and efficient means of treatment, showcasing the broader potential of neoantigen-based therapies in oncology.

## 8. Conclusions

In conclusion, neoantigens have emerged as excellent targets for immunotherapy, particularly in the realm of cancer treatment. Presented as peptides on cancer cells, these antigens offer a personalized approach to immunotherapy that has shown promising clinical outcomes. Despite their potential, the application of neoantigen-based cancer vaccines is hindered by substantial costs and variability in effectiveness. Therefore, integrating comprehensive bioinformatics tools with clinical strategies will be critical for optimizing the development and application of neoantigen-based therapies. Future research in this field should focus on refining these tools and expanding the scope of shared neoantigens to enhance the accessibility and effectiveness of cancer vaccines. Ultimately, the continued exploration of neoantigen and TAA-based strategies holds significant promise for improving the prognosis of cancer patients through personalized immunotherapy.

## Figures and Tables

**Table 1:** Open source neoantigen prediction pipeline.

Name	Short Description	Format of Input	Ref.
ImmuneMirror	Integrative Pipeline and Web Server	DNA, RNA-seq	[52]
nextNEOpi	Comprehensive pipeline for neoantigen prediction from raw sequencing data	DNA, RNA-seq	[51]
Seq2Neo	A one-stop solution for neoepitope features prediction	DNA, RNA-seq	[50]
QBRC	neoantigen calling pipeline from somatic mutation	DNA, RNA-seq	[49]
pVACtools	A Computational Toolkit, including pVACseq, pVACfuse, pVACviz and pVACvector	VCF	[48]
NeoFuse	Gene fusion-derived neoantigen	RNA-seq	[47]
ASNEO	Alternative Splicing-derived neoantigen	RNA-seq	[46]

**Table 2:** Open Source prediction tools for MHC-peptide binding.

Name	MHC Class	Short Description	Ref.
DeepMHCI	I	An anchor position-aware deep interaction model, with a great performance on non-9-mer peptides	[62]
LightMHC	I	A Light Model for pMHC Structure Prediction with Graph Neural Networks	[63]
PepPPO	I	Characterize binding motif via generating repertoires of peptides presented by given MHC-I alleles	[64]
DeepMHCII	II	A novel binding core-aware deep interaction model for accurate MHC-II peptide binding affinity prediction	[65]
AEGIS	II	Apply natural language processing algorithms to identify the MHCII immunopeptidome in humans and a preclinincal mouse model	[66]
TLimmuno2	II	A transfer learning-based, long short-term memory model	[67]

**Table 3:** Open Source prediction tools for TCR-pMHC interaction.

Name	Short Description	Ref.
TABR-BERT	A BERT-based transfer learning method	[82]
TEIM	TCR–Epitope Interaction Modelling at Residue Level predicted both pairwise residue distances and contact sites involved in the TCR–epitope interactions	[80]
PanPep	Pan-Peptide Meta Learning by combining the concepts of meta-learning and the neural Turing machine, particularly when confronted with unseen epitopes	[81]
TEPCAM	TCR-Epitope identification based on Cross-Attention and Multi-channel convolution	[83]
TCRmodel2	An adapted AlphaFold framework for speedy, accurate modeling of both TCR–pMHC complexes and unbound TCRs	[84]
STAPLER	TCR-peptide specificity prediction from full-length TCR-peptide data	[85]
TCRconv	Using contextualized motifs to predicte recognition	[86]
TEINet	A deep learning framework utilizes transfer learning model	[87]
catELMo	Predicting binding between immune cells receptors and antigens based on protein sequence data	[88]
epiTCR	A Random Forest-based method dedicated to predicting the TCR-peptide interactions	[89]
MixTCRpred	A deep learning TCR-epitope interaction predictor	[90]
EPIC-TRACE	A new machine learning model that utilizes the both α and βTCR chains, epitope sequence, and MHC	[91]
TCRdock	Structural based prediction of TCR epitope specificity	[92]
DeepTR	Pan peptide-MHC class I binding prediction with a user-friendly web service	[93]
PiTE	A binding affinity prediction consists of two sequence encoders and a stack of linear layers	[94]
TPBTE	A model based on convolutional Transformer for Predicting the Binding of TCR to Epitope	[95]
AttnTAP	The bi-directional long short-term memory model for robust feature extraction of TCR sequences	[96]
PhyAugmentation	The deep neural network with physical modeling and data-augmented pseudo-labeling	[97]
ATM-TCR	Multi-head self-attention mechanism to capture biological contextual information	[98]

**Table 4:** Selected key clinical trial of neoantigens vaccines.

Trial (Format)	Cancer Type	Phase	Short Description	Ref.
NCT01970358 (SLP)	Melanoma	I/Ib	One of the pioneering works on personalized therapeutic neoantigens, demonstrating anti-tumor efficacy in combination with ICIs	[3]
NCT02035956 (mRNA)	Melanoma	I	One of the pioneering works on personalized therapeutic vaccines using TAA and neoantigens	[4]
NCT02287428 (SLP)	Glioblastoma	I/Ib	Demonstrates the feasibility of therapeutic neoantigen vaccines in immunologically ‘cold’ tumors	[5]
NCT02149225 (peptides)	Glioblastoma	I	Demonstrates the feasibility of therapeutic vaccines using TAA and neoantigens in immunologically ‘cold’ tumors	[13]
NCT02897765 (SLP)	Bladder Tumors, Melanoma, Lung Cancer	Ib	In the large cohort, neoantigen-specific CD4+ and CD8+ T cell responses were observed in all vaccinated patients	[6]
NCT02956551 (Dendritic cell)	Non-Small-Cell Lung	I	Provides new therapeutic opportunities for lung cancer treatment	[7]
NCT03639714 (GRT-C901 adenovirus-based/GRT-R902 RNA-based)	Non-Small Cell Lung Cancer, Colorectal Cancer, Gastroesophageal Adenocarcinoma, Urothelial Carcinoma	I/II	Demonstrate exceptional performance in the treatment of patients with advanced metastatic solid tumors	[110]
NCT03380871 (SLP)	Non-Small Cell Lung Cancer	Ib	Personalized neoantigen vaccine with chemotherapy and anti-PD-1 as first-line treatment for non-squamous non-small cell lung cancer	[8]
NCT03897881 (mRNA)	Melanoma	IIb	Reported clinical benefit by prolonged recurrence free survival (RFS) in patients with resected melanoma comparing ICB plus neoantigen mRNA vaccine to ICB alone.	[9,111]
NCT04161755 (mRNA)	Pancreatic Cancer	I	Reported a prolonged RFS in vaccine-responder patients compared to non-responders, demonstrating that neoantigen vaccines offer effective treatment for PDAC	[10]
NCT03953235 (GRT-C903 adenovirus-based/GRT-R904 RNA-based)	Solid Tumor	I/II	Demonstrate good tolerability and initial therapeutic potential in advanced solid tumors through vaccines targeting shared neoantigens in combination with ICI	[112]
NCT04251117 (DNA)	Hepatocellular carcinoma	I/II	Demonstrate personalized therapeutic vaccines enhancing responses to PD-1 inhibitors through the induction of tumor-specific immunity	[113]

SLP, synthetic long peptides.

## Supplementary Materials

The following supporting information can be downloaded at: https://www.mdpi.com/article/10.3390/vaccines12070717/s1, Supplementary Table S1: Ongoing clinical trials of personalized neoantigen-based vaccines.
