# Supplementary material for: Development and Clinical Applications of Therapeutic Cancer Vaccines with Individualized and Shared Neoantigens
Source: Vaccines (Basel). 2024 Jun 27;12(7):717. doi: 10.3390/vaccines12070717 (PMC11281709; doi:10.3390/vaccines12070717)
Supplement: Supplementary file 1 [file vaccines-12-00717-s001.zip › vaccines-3057220-supplementary.pdf]

Supplementary Table S1 : Ongoing clinical trials of personalized neoantigen-based vaccines

Vaccine format	Trial	Tumour type
DC	NCT03674073	Liver Cancer
	NCT03871205	Non Small Cell Lung Cancer, Small Cell Lung Cancer
	NCT06344156	Pancreatic Cancer
	NCT04912765	Liver Cancer, Colorectal Cancer, Liver Metastases
	NCT05749627	Advanced Malignant Solid Tumors
	NCT05235607	Melanoma, Bladder Cancer, Colorectal Cancer
	NCT06342908	Diffuse Hemispheric Glioma
	NCT05767684	Solid Tumor
	NCT05773859	Ovarian Carcinoma
	NCT06329908	Lung Cancer
	NCT03914768	Diffuse Intrinsic Pontine Glioma or Glioblastoma
	NCT05023928	Diffuse Intrinsic Pontine Glioma, Diffuse Midline Glioma
	NCT05317325	Esophageal Cancer
Peptides	NCT05741242	Solid Tumor
	NCT04397926	Non Small Cell Lung Cancer
	NCT03558945	Pancreatic Cancer
	NCT05111353	Pancreatic Cancer
	NCT06195293	Solid Tumor
	NCT04487093	Non Small Cell Lung Cancer
	NCT04266730	Non Small Cell Lung Cancer, Head and Neck Cancer
	NCT06095934	Non Small Cell Lung Cancer with EGFR Gene Mutation
	NCT05269381	Breast Cancer
	NCT05557240	Glioma
	NCT05098210	Melanoma, Breast Cancer
	NCT03219450	Lymphocytic Leukemia
	NCT04087252	Cancer
	NCT04810910	Pancreatic Cancer
	NCT05307835	Esophageal Cancer
	NCT03361852	Follicular Lymphoma
	NCT03929029	Melanoma
	NCT06314087	Advanced Tumors
	NCT05475106	Neoplasms
	NCT05641545	Advanced Renal Cell Carcinoma
	NCT05444530	Myeloproliferative Neoplasms
	NCT04998474	Non Small Cell Lung Cancer
	NCT04943848	Diffuse Intrinsic Pontine Glioma, Diffuse Midline Glioma
	NCT04864379	Advanced Malignant Solid Tumor
	NCT03568058	Advanced Cancer
mRNA	NCT05192460	Gastric Cancer, Esophageal Cancer, Liver Cancer
	NCT05359354	Solid Tumor
	NCT05227378	Gastric Cancer
	NCT06195384	Solid Tumor
	NCT03908671	Esophageal Cancer, Non Small Cell Lung Cancer
	NCT06326736	Pancreatic Cancer
	NCT06019702	Digestive System Neoplasms
	NCT06141369	Adrenal Cortical Carcinoma, Medullary Thyroid Cancer, Thymic Neuroendocrine Carcinoma, Pancreatic Neuroendocrine Tumor
	NCT06026800	Digestive System Neoplasms
	NCT06026774	Digestive System Neoplasms
	NCT06156267	Pancreatic Cancer
	NCT05198752	Solid Tumor
	NCT06353646	Pancreatic Cancer
	NCT05940181	Solid Tumor
	NCT05916261	Advanced Pancreatic Cancer
	NCT05916248	Advanced Solid Tumor
DNA	NCT05743595	Unmethylated Glioblastoma
	NCT04015700	Glioblastoma
	NCT04397003	Extensive-stage Small Cell Lung Cancer
	NCT05078866	Lynch Syndrome
